# Comparing cryotherapy and ketorolac tromethamine against room-temperature saline irrigation using interleukin-8 levels and post-operative pain within single-visit endodontic treatment of symptomatic irreversible pulpitis superimposed by apical periodontitis; a comparative randomized controlled trial

**DOI:** 10.1186/s12903-025-07347-7

**Published:** 2025-12-12

**Authors:** Yousra Khaled Ezzat, Alaa Diab, Olfat Shaker, Sarah Abouelenien

**Affiliations:** 1 Department of Endodontics, Faculty of Dentistry, Cairo University, Cairo, Egypt; 2 Department of Medical Biochemistry and Molecular Biology, Faculty of Medicine, Cairo University, Cairo, Egypt

**Keywords:** Cryotherapy, Ketorolac Tromethamine, Room-temperature saline, Irreversible pulpitis with apical periodontitis, Interleukin-8, Post-operative pain, Single-visit endodontics

## Abstract

**Objectives:**

This comparative randomized controlled trial aimed to compare Cryotherapy and Ketorolac Tromethamine (KT) against a control group of Room-Temperature Saline (RtS) irrigations using Interleukin-8 (IL-8) expression and pain-intensity scale in single-visit endodontic treatment of symptomatic irreversible pulpitis superimposed by apical periodontitis.

**Materials and methods:**

Forty-eight patients diagnosed with symptomatic irreversible pulpitis superimposed by apical periodontitis, were randomly assigned into three equal groups (*n* = 16). First group was irrigated using Cryotherapy (2.5 °C saline), second group was irrigated using KT and third group was irrigated using RtS (control). Pulp Chamber Blood samples were collected before irrigation (S1) and periapical fluid samples were collected after completion of irrigation (S2) and analyzed using enzyme-linked immunosorbent assay (ELISA) for IL-8 levels. Pain was assessed pre-operatively, at 6, 12, and 24 h after obturation using a modified visual-analogue scale.

**Results:**

No significant differences were found between groups regrading pre-irrigation IL-8 levels or pre-operative pain. Post-irrigation, IL-8 levels showed a significant increase with KT than the other groups. At 6 h post-operatively, KT presented significantly lower pain scores than the other groups. No significant differences were observed at 12–24 h regarding pain scores.

**Conclusion:**

Although intra-canal irrigation using KT presented higher levels of IL-8 post-irrigation; it significantly reduced post-operative pain at 6 h following obturation. Cryotherapy reported no significant differences than RtS regarding IL-8 expression or pain levels.

## Introduction

Irreversible pulpitis necessitates complete pulpal removal for resolution (Lapidus et al., 2016) [1] While pulpectomy effectively mitigated endodontic pain, studies reported a post-operative pain ranging from 3% to 58% followed single-visit root canal therapy (Sathorn et al., 2008) [2]. Patients with severe pre-operative pain exhibited a propensity for greater post-operative discomfort, highlining a critical need for effective management (Sethi et al., 2014) [3].

Histological evaluations revealed a significant infiltration of immune cells into the inflamed pulpal tissues (Kim et al., 1992; Izumi et al., 1995) [4, 5]. Those immune cells elicited a robust inflammatory response, characterized by the production of pro-inflammatory cytokines as interleukin-6 (IL-6), interleukin-8 (IL-8), and tumor necrosis factor-alpha (TNF-α). Furthermore, those cytokines activated sensory nerve terminals and induced nociceptive pain (Hahn et al., 2001) [6]. IL-8 and TNF-α were produced by mast cells to recruit and activate neutrophils and T lymphocytes (Theoharides and Cochrane, 2004; Galli et al., 2005) [7, 8]. Substance P (SP) enhanced IL-8 production.

Recent studies compared Ketorolac Tromethamine and Dexamethasone as root canal irrigants revealed that Dexamethasone resulted in significantly lower post-operative pain and reduced analgesic requirements, whereas Ketorolac Tromethamine demonstrated superior control over expression of SP and IL-8 (Evangelin et al., 2019) [9].

Additionally, cryotherapy has been shown to reduce post-operative pain through application of cold saline irrigation that induced vasoconstriction and decreased tissue permeability, thereby diminished the release of pain mediators and limited the exudative escape in the peri-radicular tissues (Vera et al., 2018) [10]. Moreover, cryotherapy caused retardation of neural pain signal propagation (nerve conduction velocity in nociceptive sensory fibers), the phenomena was described as “cold-induced neuropraxia” (Nadler et al., 2004; Algafly and George, 2007; Herrera Villabona et al., 2010) [11–13]. The current study aimed to compare the effectiveness of Cryotherapy and Ketorolac Tromethamine against a control group of Room Temperature Saline irrigation during single-visit endodontic treatment within mandibular molars suffered symptomatic irreversible pulpitis superimposed by apical periodontitis.

## Materials & methods

### Study design and groups formulation

**Fig. 1:**
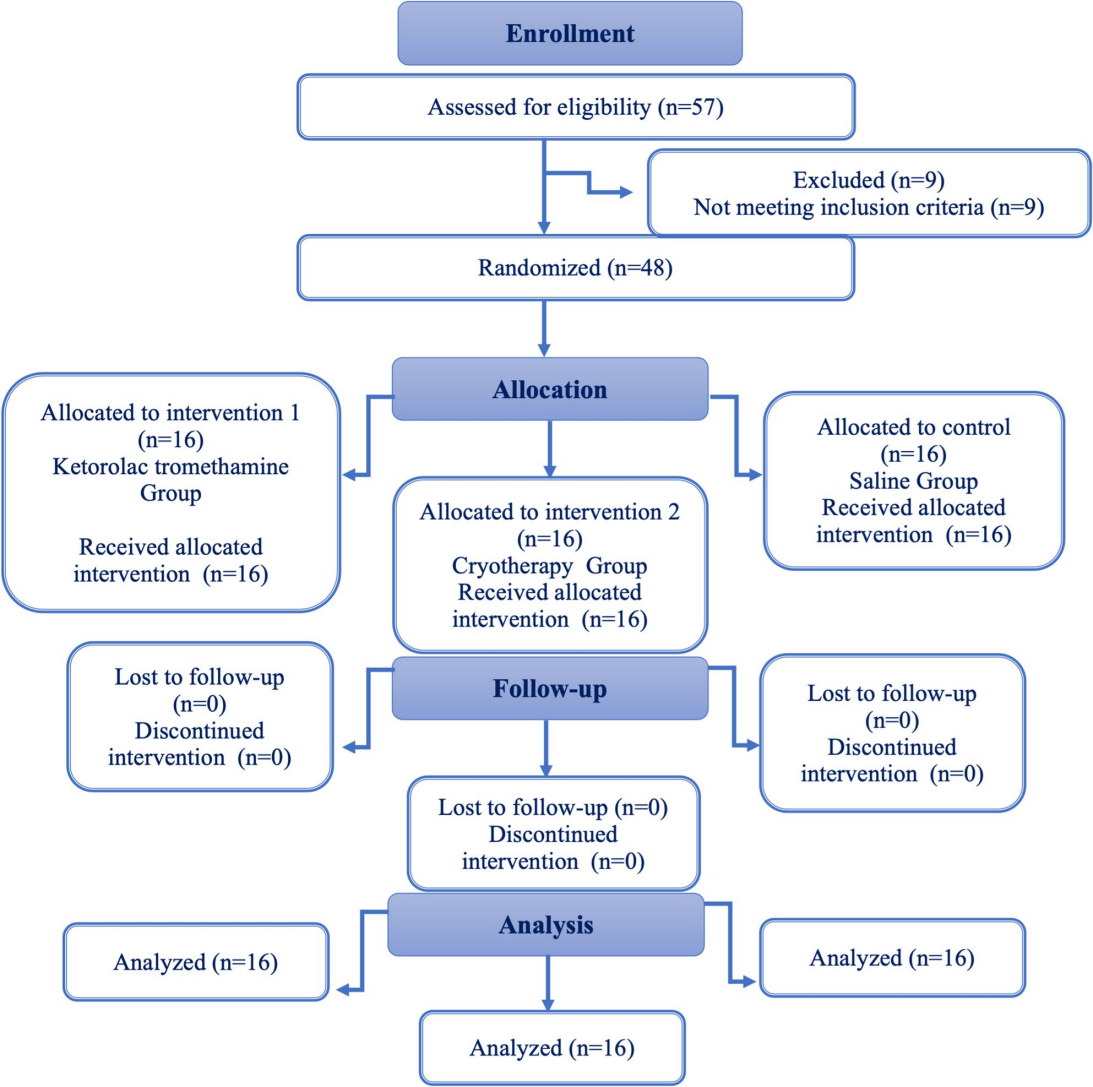
CONSORT 2010 flow diagram of the trial design

- Ethical considerations and study registration: This trial received ethical approval from the Research Ethics Committee of the Faculty of Dentistry, Cairo University. Written informed consent was obtained from all participants after a thorough explanation of the study’s objectives, procedures, benefits and risks. The trial was registered at ClinicalTrials.gov (Identifier: NCT04733326); registration date (24/01/2021) and reported in accordance with the Consolidated Standards of Reporting Trials (CONSORT) guidelines (Schulz et al., 2010) [14].
- Participant selection: Participants were recruited from the outpatient clinic of the Department of Endodontics, Faculty of Dentistry, Cairo University, between September 2021 and March 2023.
- Inclusion criteria: Participants were systemically healthy (ASA I) and diagnosed with symptomatic irreversible pulpitis with apical periodontitis (moderate to severe lingering pain lasted more than 1 min with cold testing superimposed by pain on percussion). Teeth involved were mandibular molars, with age range set to be between 20 and 40 years old.
- Exclusion criteria: Cases on use of recent analgesics or medications affecting pain perception, presence of acute periapical or periodontal abscesses or mobility greater than grade 1 all were excluded.
- Enrollment: Of the 57 patients assessed for eligibility, 48 subjects met the inclusion criteria and were enrolled in the study. All participants completed the study and were included into the final analysis.
- Randomization and blinding: Randomization was generated using an online tool (http://www.random.org/). Allocation concealment performed using numbered, opaque, sealed envelopes into three equal groups and the list was kept confidential by a co-supervisor not involved in treatment (Fig. 1). The blinding of data to avoid any bias involved patients, statistician, treating endodontist and the ELISA specialist. The patients received rubber dam isolation with high suction tip so they did not feel the temperature of the irrigant. Another colleague doctor -irrelevant to the study- received the irrigation syringes from the co-supervisor and performed the irrigation step only; hence the operator could not know the solution and the blinding was safe.

### Clinical procedure and sampling according to timeline

**Fig. 2:**
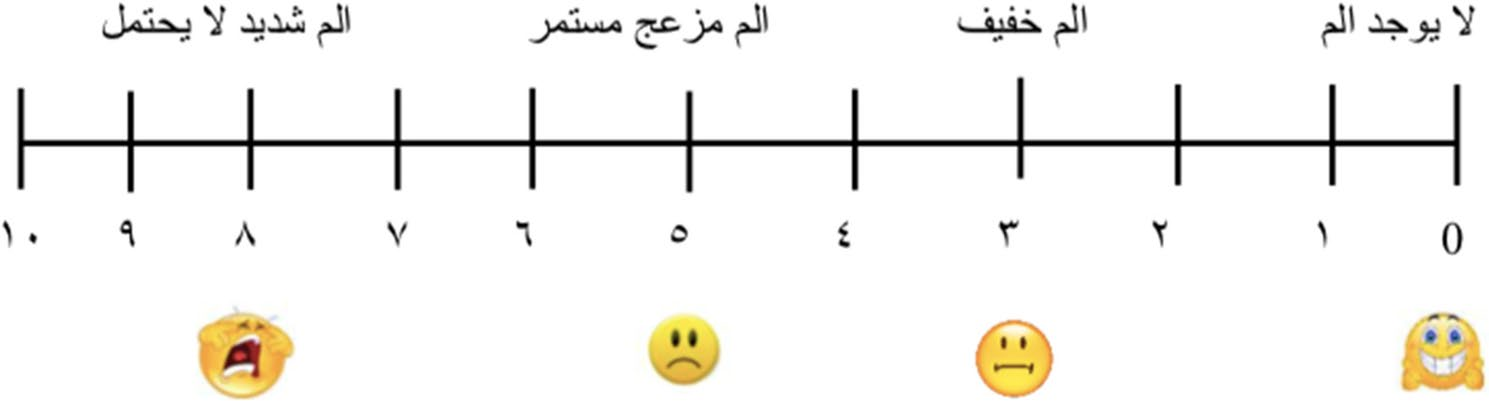
VAS pain scale
- Anesthesia^1^ was provided and access cavity preparations were completed under complete rubber dam isolation.
- Pulp chamber blood samples (S1): Those were collected using 3 sterile size-20 paper-points^2^ within each pulpal chamber for 60 s. Samples were stored in Eppendorf tubes^3^ at − 40 °C for analysis (Fig. 3).

- Preoperative pain severity assessment: That was quantified using a 10 cm Modified Visual Analogue Scale (VAS), where 0 denoted “no pain” and 10 indicated “worst imaginable pain.” Pain levels were categorized as mild (0–3), moderate (4–7), or severe (8–10) (Fig. 2).

**Fig. 3:**
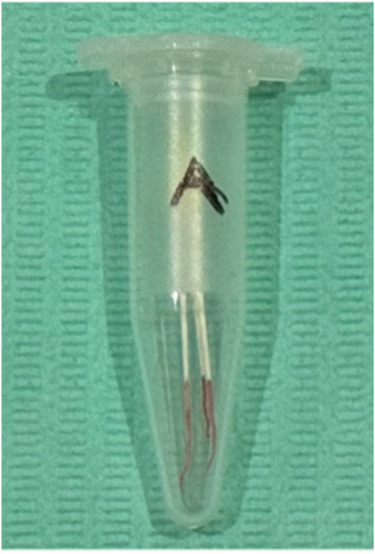
Preoperative pulp chamber blood sample S1
- Working lengths were determined and confirmed radiographically.
- Initial negotiation utilized #10 and #15 K-files^4^ followed by the crown-down technique with ProTaper Next rotary files up to size X3 (30/0.07 taper), except for distal roots (single large canals) which were prepared up to size 40/0.06 taper (X4)^5^.
- Irrigation using a sterile 27-gauge side-vented needle^6^ 1 mm short of the working length. Canals irrigated with 5 mL of 2.5% sodium hypochlorite (2.5% NaOCl)^7^ between each instrument. Smear layer removal was achieved with 5 mL of 17% ethylenediaminetetraacetic acid^8^ (17% EDTA), followed by a final rinse with 10 mL of distilled water. The root canals were dried using sterile paper points until complete dryness was achieved and no traces of blood, exudate or irrigant remained.
- **C**omparative irrigation protocols:
  - Group A (Ketorolac Tromethamine)^9^** (***n* = 16): 2 ml of 30 mg concentration.
  - Group B (Cryotherapy) (*n* = 16): 30 ml of 2.5 °C cold saline performed in six 5 mL increments using a sterile 27-gauge side-vented needle (Steri Irrigation Tips; Diadent, Korea) positioned 1 mm short of the working length.
  - Group C (Control) (*n* = 16): 30 ml of room temperature saline performed in six 5 mL increments using a sterile 27-gauge side-vented needle (Steri Irrigation Tips; Diadent, Korea) positioned 1 mm short of the working length.
  - Sterile paper points were used to completely dry the canals until no blood, exudate, or irrigant solutions was evident.
- Periapical fluid samples (S2): Collected using three sterile size 20 paper points inserted 1–2 mm beyond the apex for 60 s and stored at − 40 °C for subsequent analysis (Fig. 4).

**Fig. 4:**
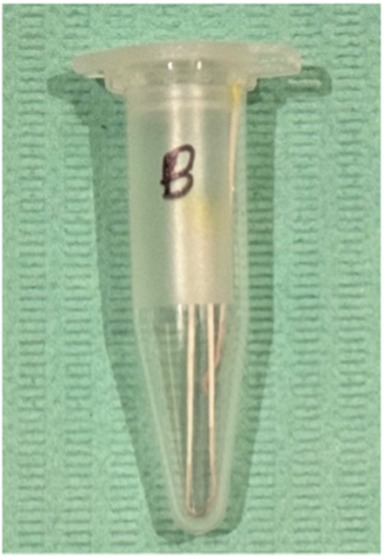
Post-instrumentation periapical fluid sample S2
- Obturation was performed using a modified single cone technique with gutta-percha cones^10^ corresponding to the master apical file size and a resin-based sealer^11^.
- Cavities were restored with glass ionomer cement with occlusal reduction to minimize post-operative discomfort.
- Post-operative pain severity assessment:
  - Post-operative pain was quantified using a 10 cm Modified Visual Analogue Scale (VAS), where 0 denoted “no pain” and 10 indicated “worst imaginable pain.” Pain levels were categorized as mild (0–3), moderate (4–7), or severe (8–10).
  - Participants were instructed to record pain intensity at 6, 12, and 24 h post-treatment and received no analgesics. On the following day, charts were collected from them and provided with Ibuprofen 400 tabs three times per day as needed.
- Elisa determination of IL-8:
  - The IL-8 level was measured using Human Interleukin-8 (IL-8) Elisa Kit^12^ using Sandwich-ELISA method. The kit was provided by SunLong Biotech Co., LTD (Catalogue Number: SL3409Hu, China). The Micro ELISA strip plate was pre-coated with an antibody specific to IL-8.
  - The protein under investigation was immobilized on a test surface.
  - A blocking agent was added to avoid undesirable attachments of other proteins.
  - Horseradish Peroxidase (HRP) - conjugated antibody specific for IL-8 was added to apparatus wells and samples were dipped into the wells.
  - Samples were incubated to allow for desired protein to attach to the antibody.
  - Unattached materials were washed-out.
  - TMB substrate solution (A detecting antibody) was added to each well. Only those wells that contain IL-8 and HRP conjugated IL-8 antibody would appeared blue in color and then turned yellow after addition of the stop solution.
  - The optical density (OD) was measured spectrophotometrically at wavelength of 450 nm. The OD value was proportional to the concentration of IL-8. The concentration of IL-8 was calculated by comparing OD of samples to the standard curve using a specific-reader and results were interpreted, ELISA (Figs. 5, 6 and 7).

**Fig. 5:**
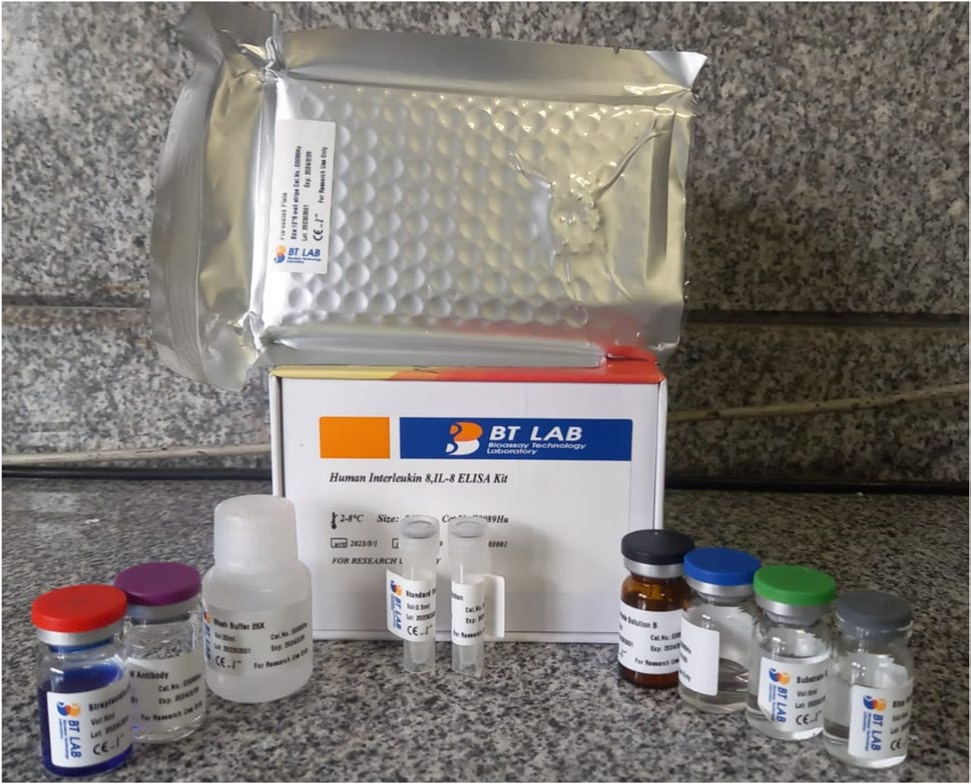
ELISA kit

**Fig. 6:**
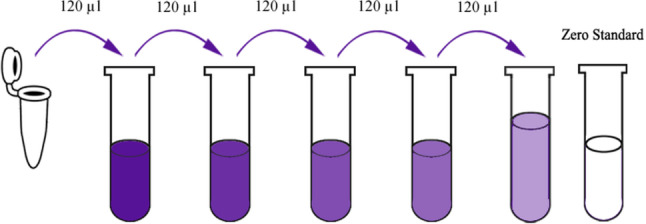
ELISA reagent preparation

**Fig. 7:**
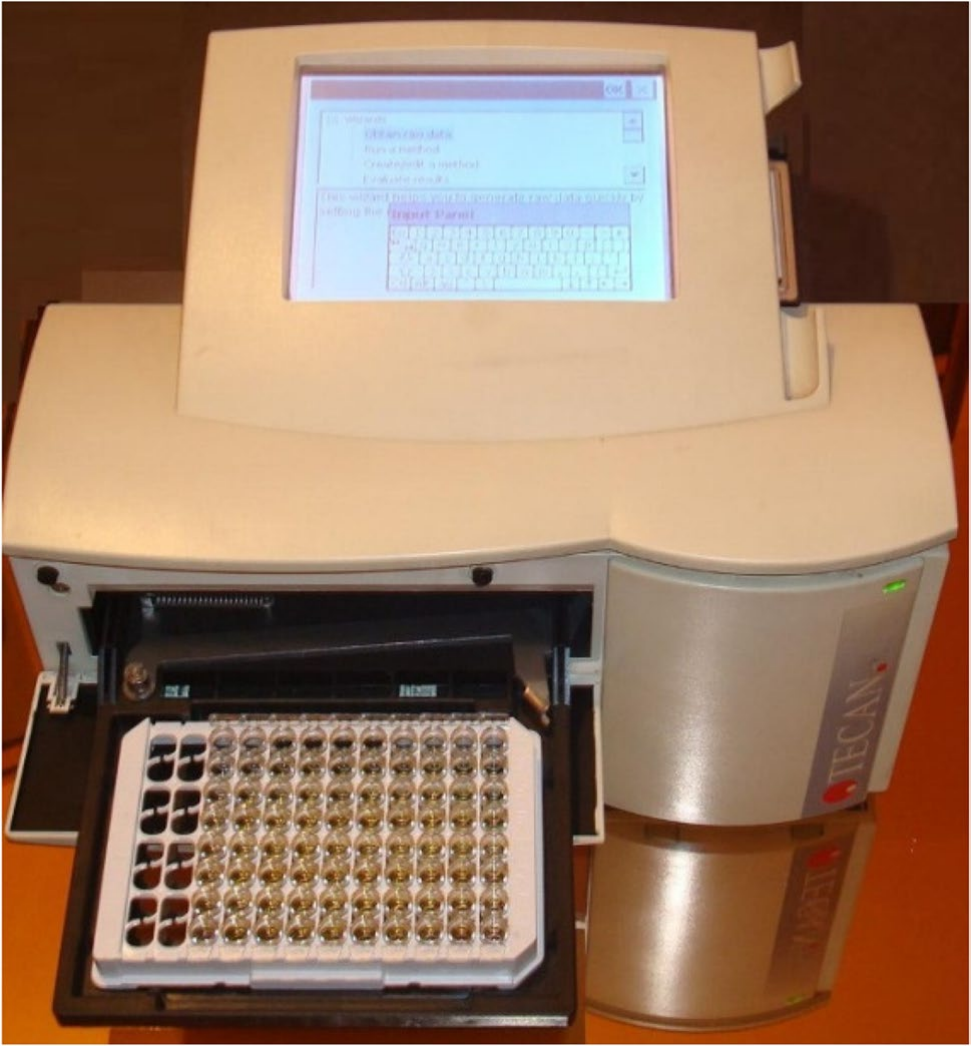
ELISA reader

### Statistical analyses

Statistical analyses were conducted using IBM SPSS Statistics for Windows, V23.0 (IBM Corp., Armonk, NY, USA). Normality was assessed using Kolmogorov-Smirnov and Shapiro-Wilk tests. One-way ANOVA compared age across groups, with repeated measures ANOVA and Bonferroni post-hoc tests compared IL-8 levels across groups. Kruskal-Wallis and Friedman tests analyzed VAS scores between and within groups, with Dunn’s test for post-hoc comparisons. Spearman’s correlation coefficient assessed the relationship between IL-8 and pain scores. Categorical variables were analyzed using Chi-square test.

## Results

Age and IL-8 exhibited normal distribution. VAS scores were non-parametric. Parametric data were expressed as mean ± standard deviation (SD), and non-parametric data as median and range. The mean age was of 32.8 ± 4.1 years. The gender distribution was balanced across groups, with 23 males (47.9%) and 25 females (52.1%). Mandibular molars were evenly represented: 24 (50%) were first molars and 24 (50%) were second molars. Baseline demographic and clinical characteristics, including age, gender, and tooth type, were statistically comparable among the three groups (*p* > 0.05) (Table 1).

**Table 1:** Frequencies (*n*), percentages (%), mean and standard deviation (SD) values for base line characteristics in the three groups

	Base line characteristics		Ketorolac (*n* = 16)	Cryotherapy (*n* = 16)	Saline (*n* = 16)	*P*-value
Gender	Male	*n*, (%)	8 (50%)	6 (37.5%)	9 (56.2%)	0.557
	Female	*n*, (%)	8 (50%)	10 (62.5%)	7 (43.8%)	
Age in years		Mean, (SD)	31.7 (4.5)	34.4 (4.0)	32.3 (3.4)	0.147
Tooth Type	First molar	*n*, (%)	7 (43.8%)	7 (43.8%)	10 (62.5%)	0.472
	Second molar	*n*, (%)	9 (56.2%)	9 (56.2%)	6 (37.5%)	

* Significant at *P* ≤ 0.05

### Pain intensity

**Fig. 8:**
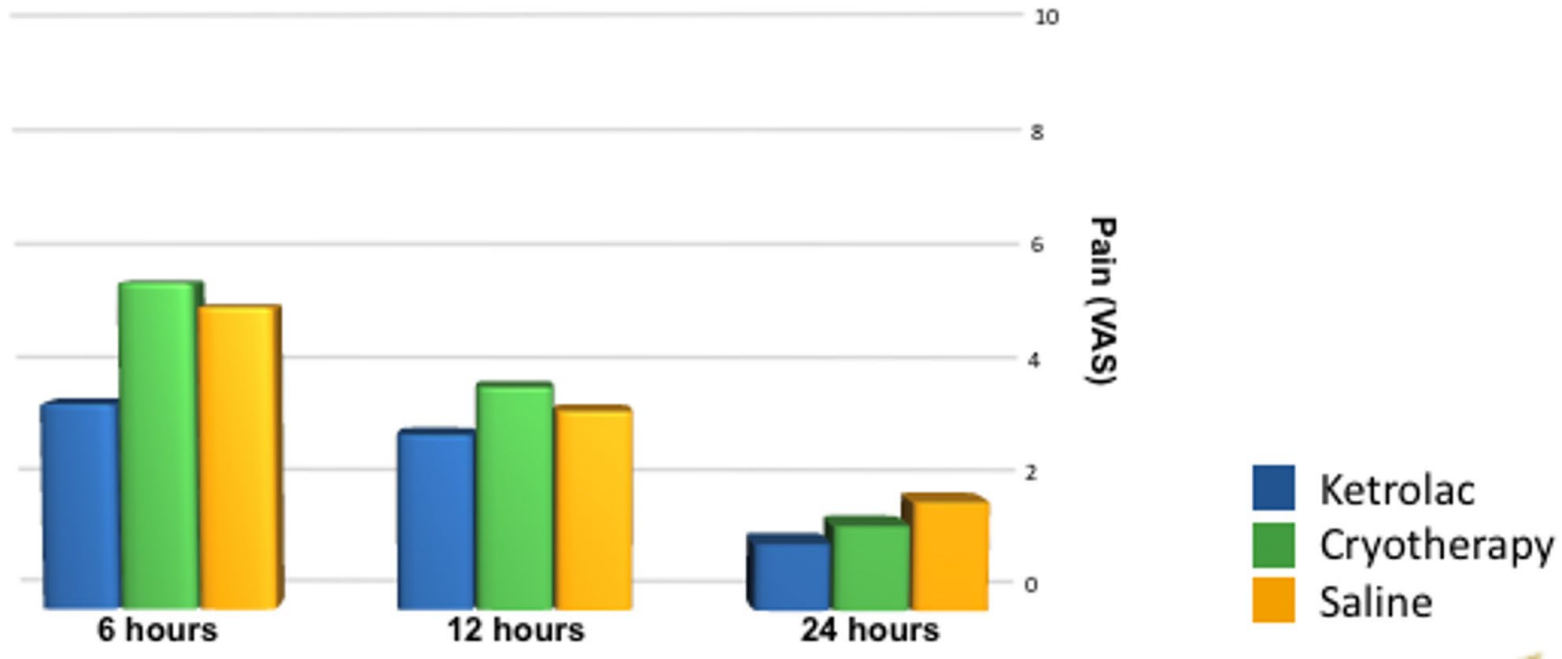
Bar chart representing mean values of the intensity of post-operative pain (VAS scores)

Pre-operative and immediate post-operative pain was nonsignificant between the groups. At the 6-hour time point, the KM group reported significantly lower mean than Cryotherapy and RtS groups. However, at 12 and 24 h, the differences between groups were not statistically significant (*p* > 0.05) (Fig. 8)

### Interleukin-8 levels (IL-8)

**Fig. 9:**
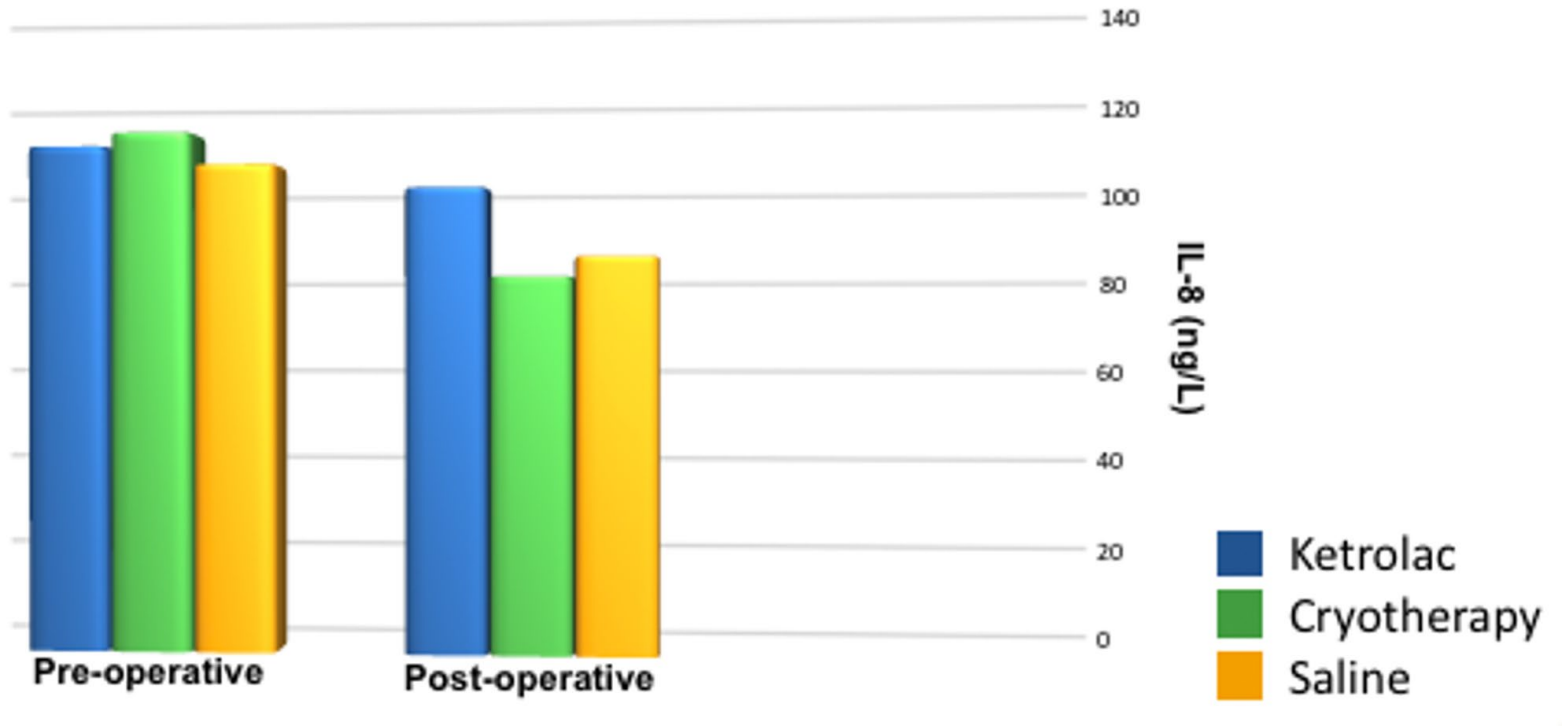
Bar chart representing mean values of pulpal interleukin-8 levels (ng/L)

No significant differences detected between the groups regarding IL-8 levels before irrigation. A significant overall reduction in post-treatment concentrations than baseline IL-8 levels (*p* = 0.027). Intergroup comparisons showed that KM group exhibited higher mean post-treatment than Cryotherapy and RtS groups. The difference between Cryotherapy and RtS groups was not statistically significant (Fig. 9)

## Discussion

This randomized comparative controlled clinical trial was designed to compare the efficacy of Cryotherapy and Ketorolac Tromethamine against room-temperature saline using post-operative pain intensity and IL-8 expression within cases of symptomatic irreversible pulpitis superimposed by apical periodontitis. Mandibular molars were selected in trial due to their high caries susceptibility. Previously published studies had shown that 59.8% of mandibular molars suffer from caries immediately after eruption as compared to 27.9% for maxillary molars (Demirci et al. 2010; Hamaghareeb et al., 2023) [15, 16].

### Sample size determination

Sample size determination was based on a power analysis used post-operative pain intensity measured via the Visual Analogue Scale (VAS) at 24 h. Reference data from (Gundogdu and Arslan, 2018) [17] indicated a mean VAS score of 77.76 ± 19.534 in the control group. Assumed an anticipated mean difference of 25 units between experimental and control groups and applied alpha level of 0.05 and beta level of 0.20 (power = 80%), the minimum required sample size was calculated to be 11–16 participants per group. To account for an estimated dropout rate of 15%, each group was expanded to include 16 participants, yielded a total sample size of 48 subjects. Sample size calculation was performed using PS Power and Sample Size Calculations software V3. (Hawker et al., 2011; Aggarwal et al., 2012; Fowler et al., 2014) [18–20].

Of the 57 patients initially screened for eligibility, 48 participants (*n* = 16 per group) met the inclusion criteria and were enrolled in accordance with the CONSORT guidelines. Randomization yielded homogenous groups with respect to baseline characteristics including age, gender distribution, tooth type, and preoperative pain levels. The absence of statistically significant differences in these variables indicated successful allocation concealment and balanced distribution of potential confounding factors across the study arms (Suresh et al., 2011 and Somaraj et al., 2016) [21, 22]. This aligned with the findings of Sadaf et al. 2014 [23].

### Interluekin-8 and ELISA testing

The idea was formed as adding a selective antibody that bonded to the protein under investigation. Later on, after washing all un-necessary proteins, detecting agent was added to join protein-antibody complex to be detected through fluorescence of special colors. Interleukin (IL-8):The pre-operative pulpal IL-8 levels revealed non-statistically significant differences amongst all groups. After irrigation, pulpal IL-8 levels were higher with KM than the other groups. It is conceivable that the effect of Ketorolac was more toward pain reduction than on inflammation subsiding ability. On the other hand, cryotherapy produced similar IL-8 levels as RtS, which indicated that no demonstratable effect of temperature on inflammatory process reduction. Those findings were in accordance with (Keskin et al., 2017; Emad et al., 2021) [24, 25].

### Pain intensity and the visual analogue scale (VAS)

Previously published studies speculated that the use of intra-canal cryotherapy boosted the efficacy of pulpal anesthesia and reduced the post-operative pain scores after single visit endodontic treatment (Keskin et al., 2017; Vera et al., 2018; Hespanhol et al., 2022) [10, 24, 26]. Moreover, it had been documented that the application of NSAIDs locally inside the canals was effective in reducing the inter-appointment and post-operative pain (Yapp et al., 2013; Coll and Geddes, 2022; Uysal et al., 2022) [27–29]. In this study, KM demonstrated a significant decline in pain intensity scores at 6 h post-operative than cryotherapy and RtS. On the other hand, there was no significant difference at immediate post-operative pain, 12–24 h post-treatment between the three groups. This meant that cryotherapy did not provide any effect on pain management than room temperature saline, contrary to previously published studies, where the use of intra-canal cryotherapy reduced post-operative pain more than room temperature saline (Sadaf et al., 2020; Hespanhol et al., 2022) [26, 30].

Further randomized clinical trials with larger sample sizes along with expanded cytokine profiling is recommended to compare more precisely the impact of intra-canal NSAIDs on the incidence of post-operative pain as well as the levels of pro-inflammatory cytokines.

## Conclusion

- Intra-canal irrigation using Ketorolac Tromethamine (KM) effectively reduced the post-operative pain after 6 h of treatment in teeth suffered from acute pulpitis super-imposed with apical periodontitis. On the contrary, it presented with significant higher levels of Interleukin-8 (IL-8) inflammatory mediator after irrigation than cryotherapy and room temperature saline (RtS).
- No other significant differences were noticed between the irrigation with Ketorolac Tromethamine (KM), cryotherapy and room temperature saline (RtS).

### Study limitations

- Sample size, while statistically justified, could be expanded for subgroup analyses.
- Inclusion of longer follow-up intervals and multi-cytokine profiling is recommended for future research.

## Data Availability

The datasets generated and analyzed during the current study are available from the corresponding author on reasonable request.

## Footnotes

1 Articaine HCl 4% and Epinephrine 1:100,000.

2 Meta Biomed Co, Ltd, Korea.

3 Suzhou Bioselec Biotechnology Co., Ltd. China.

4 K-Files, MANI, INC., Industrial Park, Utsunomiya, Tochigi, Japan.

5 Dentsply Maillefer, Ballaigues, Switzerland.

6 Steri irrigation tips; Diadent, Chungcheongbuk-do, Korea.

7 Dent House, Medical company for household products, Cairo, Egypt.

8 Meta biomed Co.,Ltd, Korea.

9 Amriya Pharmaceutical Industries, Alexandria, Egypt.

10 Meta Biomed Co., Ltd, Korea.

11 Adseal, Meta Biomed CO.,LTD, Korea.

12 Human IL-8 ELISA KIT provided by Bioassay Technology with Cat.No E0089Hu, China.
